# Improving Prediction of Springback in Sheet Metal Forming Using Multilayer Perceptron-Based Genetic Algorithm

**DOI:** 10.3390/ma13143129

**Published:** 2020-07-14

**Authors:** Tomasz Trzepieciński, Hirpa G. Lemu

**Affiliations:** 1 Department of Materials Forming and Processing, Rzeszow University of Technology, al. Powst. Warszawy 8, 35-959 Rzeszów, Poland; tomtrz@prz.edu.pl; 2 Faculty of Science and Technology, University of Stavanger; N-4036 Stavanger, Norway

**Keywords:** elastic strain, genetic algorithm, material properties, perceptron-based prediction, springback, steel sheet metal

## Abstract

This paper presents the results of predictions of springback of cold-rolled anisotropic steel sheets using an approach based on a multilayer perceptron-based artificial neural network (ANN) coupled with a genetic algorithm (GA). A GA was used to optimise the number of input parameters of the multilayer perceptron that was trained using different algorithms. In the investigations, the mechanical parameters of sheet material determined in uniaxial tensile tests were used as input parameters to train the ANN. The springback coefficient, determined experimentally in the V-die air bending test, was used as an output variable. It was found that specimens cut along the rolling direction exhibit higher values of springback coefficient than specimens cut transverse to the rolling direction. An increase in the bending angle leads to an increase in the springback coefficient. A GA-based analysis has shown that Young’s modulus and ultimate tensile stress are variables having no significant effect on the coefficient of springback. Multilayer perceptrons trained by back propagation, conjugate gradients and Lavenberg–Marquardt algorithms definitely favour punch bend depth under load as the most important variables affecting the springback coefficient.

## 1. Introduction

Bending is a method of plastic working consisting in plastic deformation of the materials under the influence of the bending moment. Elastic deformation of the material resulting from the relaxation of the elastic part of internal stresses after unloading is one of the basic phenomena determining the dimensional and shape accuracy of formed elements [1,2]. The phenomenon of changing the geometry of bent products, which often have complex geometry, is also the result of the nonuniformity of the state of deformation on the cross-section of the bent element. The bent element is conditioned by the mechanical properties of the material (e.g., yield stress, tendency to work hardening, microstructure and anisotropy of mechanical properties) and the geometric parameters of the bending, including bending radius, the ratio of width to thickness of the sheet metal [3] and technological factors, including temperature [4] and speed of deformation [5]. The assessment of sheet metal springback is carried out in appropriate free bending tests, including the V-shape [6,7] and U-shape [1,8,9] bending, flanging [10], three- and four-point bending, cylindrical bending or rotary bending tests. However, the most commonly used experimental method to analyse sheet metal springback is V-shaped bending, which is easy to carry out. Bending is one of the metal forming processes that allows one to get the finished product within a narrow range of dimensional tolerances. Knowledge of the amount of elastic springback is necessary at the tool design stage. The idea of tool correction is to use compensation for elastic deformations by selected process parameters, the shape of the preform die or both dimensional and shape correction of the tool [11,12]. When designing the bending process, the critical bending radius should be taken into account. The value of the critical bending radius depends on the surface quality of the material being bent, the material’s tendency to crack, and the position of the bending line in relation to the rolling direction of the sheet metal.

Traditional approaches to control springback in the sheet metal industry are based on trial-and-error procedures. Among numerical techniques, the finite element method (FEM) takes precedence and is currently the most popular [13,14]. FEM simulations permit one to determine the stress and strain distribution, material deformations, forming forces and potential locations of defects in the forming process. An effect of computational parameters on springback prediction by FE-based numerical simulation has been studied by Trzepiecinski and Lemu [7]. Theoretical [15], analytical [8,14], semi-analytical [16] and methods of artificial intelligence are also widely used to predict springback behaviour. Most analytical studies have focused on air bending while limited work has been done on V-die bending due to the inherent difficulties arising from the coining action associated with this problem [6].

Artificial neural networks (ANNs) are tools that enable the construction of linear [17] and nonlinear [18] models that solve complex classification [19] and regression [20,21] tasks in many areas of industrial problems. Calculations performed by neural networks belong to the techniques of so-called soft computing. In contrast to finite element (FE) modelling, an Artificial Neural Network (ANN) is trained based on experimental results in order to make predictions regarding the elastic unloading of a workpiece. A springback prediction model has been developed by Kazan et al. [22] for the wipe-bending process by using the ANN approach. Sharad and Nandedkar [23] predicted springback by using ANN based on the FEM results of U-bending, for various die radii, sheet thicknesses, strain hardening exponents and strength coefficients, and for two different materials. The results obtained by numerical models compared with ANN are found in good agreement. Jamli et al. [24] used FE program integrated with the ANN to facilitate nonlinear elastic recovery of sheet metals in the draw-bend test. The results show the significance of emphasising the nonlinearity of the unloading modulus instead of the chord modulus, as the final product of springback will result with residual stresses. Panthi et al. [25] used ANN springback predictions to investigate the effect of velocity on springback in a straight flanging process. Song and You [26] investigated the prediction of springback in the bending process of a T-section beam using ANN and FEM. The FE-based simulation results of springback show satisfactory agreement with the experimental data. The maximum relative error was less than 10%. Liu et al. [9] implemented genetic algorithms (GAs) to optimise the weights of a neural network for predicting springback in typical U-shaped bending. The established GA-ANN model is capable of accurately predicting springback with less time and better convergence. 

Han et al. [27] also investigated a coupled FEM and ANN technique and a FEM with a particle swarm optimisation (PSO) algorithm, which was used to optimise the weights and thresholds of the ANN model. It was observed that the BP neural network prediction model of the proposed PSO algorithm improved not only the accuracy of prediction, but also the learning speed of the network. Şenol et al. [28] used ANN to predict the amounts of springback of stainless steel sheets through experiment-based networks. It was concluded that ANN can be effectively applied to determine springback in the air bending process. Spathopoulos and Stavroulakis [29] used Bayesian regularised back propagation (BP) networks and FEM to predict springback in sheet metal forming processes. A good agreement was attained between the target and predicted output values. An overview of the literature on the use of ANN applications in the prediction of springback has been provided by Dib et al. [30].

The literature review has shown that most of the investigations focused on the development of regression models of the experimental data, often without proper selection of input features and without determination of their importance in the model. This paper proposes a novel coupled genetic algorithm-based ANN model to predict the springback properties of anisotropic DC04 sheets in a V-die air bending process. The approach developed is based on the different learning algorithms. Moreover, the input parameters of sheets with anisotropic properties for learning the ANN were processed by a genetic algorithm which, as far as the authors’ knowledge of the literature is concerned, has not been tried out for the purpose of springback prediction. The importance of the specific features in the ANN model is also determined.

## 2. Experimental

### 2.1. Material

The experiments were conducted on cold-rolled DC04 steel sheets of 2 mm thickness. The basic mechanical properties of the sheets tested were determined in uniaxial tensile tests conducted at room temperature according to the ISO 6892–1 standard. To characterise the material’s properties though uniaxial tensile test, specimens of steel sheets were cut at 0° and 90° orientations to the rolling direction. Three specimens were tested for each direction and the average values of each basic mechanical parameter (Table 1) were determined.

The values of the strain hardening exponent n and strain hardening coefficient K in the Hollomon equation are determined as follows:

(1) $$\sigma_{p}=K\cdot \varepsilon^{n}$$

where *σ_p_*—stress and *ε*—plastic strain are determined from the logarithmic true stress–true strain plot by linear regression.

The strain hardening exponent *n* can be determined using the following formula:

(2) $$n=\frac{dlog\sigma }{dlog\varepsilon }=\frac{d\sigma }{d\varepsilon }\frac{\varepsilon }{\sigma }$$

The anisotropic plastic behaviour of sheet metals is characterised by Lankford’s coefficient *r*, determined by the following formula:

(3) $$r=\frac{ln\frac{w}{w_{0}}}{ln\frac{l_{0}\cdot w_{0}}{l\cdot w}}$$

where *w*_0_ and *w* are the initial and final widths, while *l*_0_ and *l* are the initial and final gauge lengths, respectively.

Due to the anisotropic properties of the sheet material, the specimens for bending experiments, in the form of rectangular strips of dimension 20 mm × 110 mm, were cut along the rolling direction of the sheet and transverse to the rolling direction.

### 2.2. Method 

**Figure 1:**
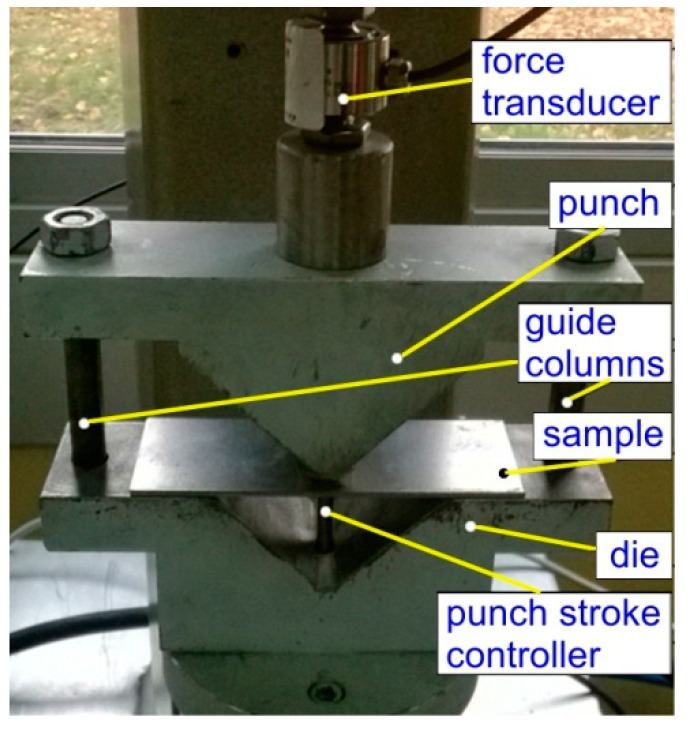
The experimental setup.

**Figure 2:**
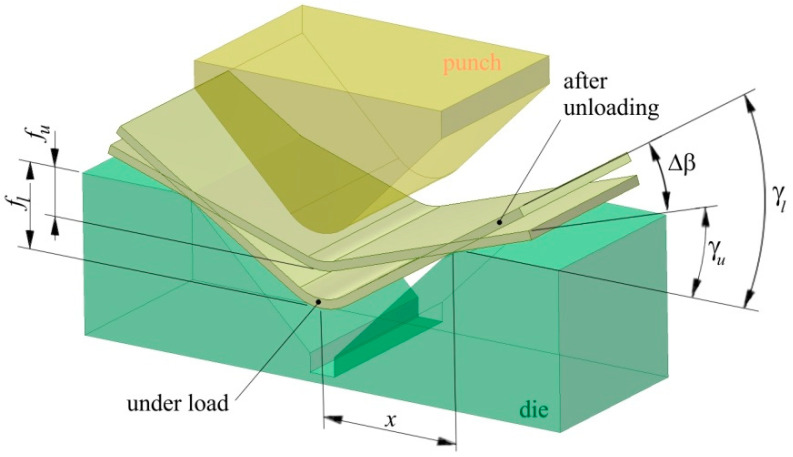
A schematic diagram of the measurement of springback.

Air bending experiments were carried out in a semi closed 90° V-shaped die (Figure 1). During the bending test, a punch bends the specimen by applying the progressive vertical movement *fl*. Punch bend depth under load *f_l_* and punch bend depth load released *f_u_* (Figure 2) were registered by the QuantumX Assistant program through punch stroke transducers. The amount of springback was then evaluated as:

(4) $$K_{s}=\frac{\gamma_{l}}{\gamma_{u}}$$

where *γ_l_* = $arctan\frac{2f_{l}}{x}$ is the bend angle under load, *γ_u_* = $arctan\frac{2f_{u}}{x}$ is the bend angle when the load is released (Figure 2). Angle Δβ defines the difference between bend angles under load *γ_l_* and after unloading *γ_u_*. For all punch depths under load, three bending tests were conducted and then the average value of springback was evaluated.

## 3. Establishment of an Artificial Neural Network Model

### 3.1. Background

Artificial neural networks are tools enabling the construction of linear and nonlinear models to solve classification and regression problems. The structure of the ANN and its operation reflects the processes occurring in the biological nervous system. They are sets of interconnected elements called neurons that process information supplied to the network’s inputs based on the idea of parallel processing. The basic feature of systems based on ANN that distinguishes them from typical information processing algorithms is their ability to generalise. It is defined as the ability of a neural network to approximate the value of a function of several variables, which is contrary to the interpolation obtainable by algorithmic processing. 

**Figure 3:**
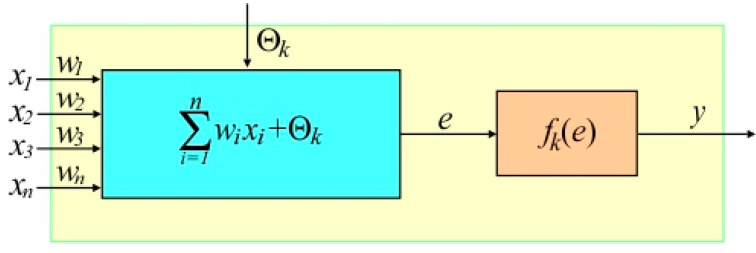
Structure of the nonlinear *k*-th neuron.

The operation of the ANNs is based on the idea of parallel processing. Each signal is introduced to the neuron through a connection of a certain weight (Figure 3). In addition, each neuron has a threshold value that determines its level of excitation. In the *k*-th neuron, the sum of the input signal values *x_n_* multiplied by the weighting factors *w_n_* is calculated, which is then increased by the bias *Θ*_k_. The stimulation *e* of the neuron is transformed by a specified activation function of the *k*-th neuron *f_k_*(*e*), which determines the nonlinear relationship between the input and output signals of the neurons. The most commonly used activation functions include the threshold function and its modifications (i.e., binary step) as well as nonlinear functions (sigmoid, logistic function, hyperbolic tangent, etc.).

Due to the network topology, unidirectional networks are distinguished in which signals flow in one direction from the input to output neurons, as are recursive networks containing feedbacks. For the architecture, single and multilayer networks are distinguished. As a result of the learning process, ANNs may acquire the ability to predict output signals based on the sequence of input signals and their corresponding output signals. The task of the learning algorithm is to select weight values and threshold values of all neurons to ensure minimisation of the error of the ANN. The surface of errors for a neural network that performs a nonlinear task is a hyper-surface with a complex shape. Starting from the initial random weight system and threshold values, the learning algorithm modifies these values in a way that aims to achieve the global minimum. During each iteration, all learning sets are presented to the network. Based on the difference in the value of the output signals with ideal cases, the value of the network error is determined.

### 3.2. ANN Modelling

The behaviour of the specific neuron and the entire ANN is strongly dependent on the type of activation function used. The basic function to activate the neurons is a threshold function. It generates a value of 0 on the neuron output if the input is less than zero or 1 if the input is greater than or equal to zero. In such conditions, it can be assumed that the neurons act in a similar fashion as biological equivalents. Subtracting the threshold value from the sum of weighted inputs is a procedure that unifies the form of the transition function. So, the output signal of neurons always occurs depending on whether the subtraction result is greater or less than zero. Moreover, the output signals of neurons do not depend on any variable of threshold value [31]. The problem with the threshold is that it does not allow multi-valued outputs. Consequently, despite the strong biological justification, the threshold activation function is rarely used in ANNs [31].

The operation of ANN when selecting a linear activation function is to directly pass a value expressing the total excitation of the neuron to its output. This type of activation function of neurons is used in different types of ANNs. However, there are some problems with a linear activation function. It does not allow to use the back-propagation algorithm to train an ANN because the derivative of a function is constant and has no relation to the input. In such conditions, all layers of the neural network collapse into one with linear activation functions, regardless of the number of layers in the ANN. The output layer is the linear function of the first layer (because the linear combination of linear functions is still a linear function) [31]. A neural network with a linear activation function works like a linear regression model with a limited ability to handle the complexity of input parameters.

Therefore, in solving complex problems using ANNs, the aim is to use the activation function providing signals with continuous changing values, although their biological interpretation is more complex than in the case with a threshold function [31,32]. Nonlinear activation functions allow the model to create complex mappings between inputs and outputs that are necessary for ANN training and modelling complex data. It was shown [32] that the amount of neuron excitation is encoded in nerve fibres by the instantaneous pulse frequency, and not by the presence or absence of a single pulse. If the frequency of impulses in a biological brain can have higher or lower values, in a certain sense, this justifies the continuous changing of signals in artificial neural networks. In this paper, the hyperbolic tangent (*tanh*) function (Equation (5)) is used as an activation function. As in the case of the logistic function, it is an S-shaped curve. Due to the bipolarity of the *tanh* activation function, it often works better than the logistic function. This function is recommended for usage in numerous nonlinear neural networks—especially in multilayer perceptrons [33,34,35].

(5) $$f_{k}\left(e\right)=\frac{e^{x}-e^{-x}}{e^{x}+e^{-x}}$$

where x is an argument (input) function.

The Multilayer Perceptron (MLP) function has been selected to model the springback phenomenon. The network learning process uses a data set containing process parameters, the mechanical properties of the test sheets, and the corresponding values of the springback coefficient *K_s_*, which was determined in the bending test. As the input signals to the network, the following parameters have been initially selected: punch bend depth under load *f_l_*, Young’s modulus *E*, yield stress *σ_y_*, ultimate tensile stress *σ_m_*, strain hardening coefficient *K*, strain hardening exponent *n*, and Lankford’s coefficient *r*. Sample orientation was not explicitly entered at the network input. However, this orientation in the input data to ANN was identified by different mechanical parameter values measured for these two orientations. The expected signal at the output is the coefficient of springback *K_s_*. The training data consist of 54 training sets. Factors affecting the amount of springback should significantly affect the elastic strains of metallic sheets and they must be independent of each other [36]. The selection of factors affecting the amount of springback is difficult due to the complex interactions of many parameters often correlated with each other.

As the magnitudes of the original training data are different, the input data must be normalised to the range (0, 1), which can be carried out using the min–max method, according to the relationship in Equation (6). Normalisation of min–max by means of a linear function transforms the values of the original data to a new range (*N_min_*, *N_max_*):

(6) $$D^{\prime }=\frac{\left(D-min\right)}{max-min}\left(N_{max}-N_{min}\right)+N_{min}$$

where (*min*, *max*) is the interval of the primary data, *D* is the value of the variable being normalised.

Among all the experimental sets of input data that correspond to the amount of springback, 20% were separated and assigned as a test set. From the remaining set of training data, 10% was separated and assigned as the validation set.

At a later stage of creation of the ANN model, the significance of individual explanatory variables was analysed. To select factors which significantly influence springback, the genetic algorithm was used. Several MLPs were built in the Statistica program for different numbers of neurons in the hidden layer. The round mean square (RMS) error values determined separately for individual subsets of data were adopted as the criterion for the assessment of the quality of training of ANNs [36]:

(7) $$RMS=\sqrt{\frac{\sum_{k=1}^{N}{\left(x_{k}-y_{k}\right)}^{2}}{N}}\;$$

where: *x_k_*—expected signal of the output neuron signal for the *k*-th pattern, *y_k_*—output signal for the *k*-th pattern, *N*—number of vectors of the training set.

Training of MLPs was carried out using several algorithms, the first of which was the back propagation (BP) algorithm. The classic BP algorithm modifies the input weight *w_ji_* by a value (Δ*w_ji_*), which is proportional to the derivative of the objective function:

(8) $$∆w_{ji}=-\eta \frac{\partial E}{\partial w_{ji}}$$

where *μ* is a learning coefficient.

During network learning by means of the BP algorithm, the direction of the greatest decrease in error is first evaluated at a predetermined point on the error surface, and then the algorithm jumps in a designated direction on the error surface to a distance proportional to the learning coefficient *μ*:

(9) $$w_{ji}\left(t+1\right)=w_{ji}\left(t\right)+\mu \delta_{j}\left(t\right)y_{i}\left(t\right)+\phi \left(w_{ji}\left(t\right)-w_{ji}\left(t-1\right)\right)$$

where *w_ij_*—input weight *i* in the *j*-th neuron, *t*—number of algorithm period, *δ_j_*—derivative of the neuron activation function, *μ*—learning coefficient, *ϕ*—coefficient of momentum.

BP training with a momentum unit allows to increase the effectiveness of the learning rate without worsening the process stability. With the coefficient of momentum *ϕ*, once the weights start moving in a particular direction in the weight space, they tend to continue moving in that direction with a certain inertia proportional to the change of this weight in the previous iteration. The use of a small value of the learning coefficient extends the calculation time, but at the same time causes precise “jumps” on the error surface to achieve the global minimum. 

The proper selection of the learning coefficient *μ* and the coefficient of momentum *ϕ* has a great effect on the rate and convergence of the learning process. Optimal values of both coefficients may be different not only for individual iterations but even for each weight. There are no analytical methods for their selection, therefore the values adopted at the beginning of the network learning process may be inappropriate. The values of these parameters should be adapted to the nature of the learning data and modified if necessary. In the investigations, the learning coefficient *μ* and coefficient of momentum *ϕ* were assumed to be 0.1 and 0.3, respectively.

Second-order algorithms, which are used to optimise nonlinear functions, i.e., the conjugate gradients (CG) algorithm, the variable metric algorithm (Quasi-Newton—QN) and the Levenberg–Marquardt (LM) algorithm [37], were also tested. According to the literature (e.g., [38]), in most applications, these algorithms are significantly faster than the BP algorithm. The algorithms cited combine the features of two groups of algorithms designed to search for the minimum of the error function: methods searching along a designated straight line and methods based on the adopted area of the model (model-trust region approach) [38]. The operation of the search algorithm along a straight line consists in guiding the straight line in a certain direction of motion on a multidimensional surface. The minimum error function is determined for all points lying on this line.

In order to prevent the overlearning of MLPs, no further reduction of the RMS error value of the validation set is assumed as the criterion for completing the network learning process. Continuing the learning process could lead to over-matching of network responses to learning vectors and loss of ability to generalise training data.

## 4. Results and Discussion

### 4.1. Experimental Springback

**Figure 4:**
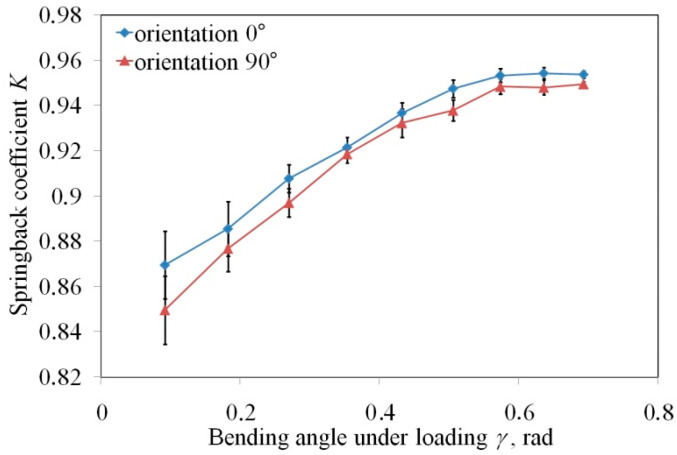
Effect of specimen orientation on the springback coefficient.

The effect of specimen orientation in the V-die bending process on the springback coefficient Ks is shown in Figure 4. As can be observed in this figure, the specimens cut along the rolling direction exhibit higher values of the springback coefficient compared to the specimens cut transverse to the rolling direction. However, the difference between the springback coefficient measured for both directions does not exceed 2.3%. The greatest difference is clearly visible for small values of bending angle under load. An increase in the bending angle γ_l_ leads to an increase in the springback coefficient. This relationship is nonlinear. According to Equation (4), low punch bend depth under load f_l_ produces high values of bending angle γ_l_. In the case of small punch depths, the material mainly undergoes elastic strains. Significant plastic deformation appears in the external layers of the sheet strips with increasing punch depth. The stress forces on the border between the elastic region and the plasticised region reach the yield stress value. So, if the material undergoes greater plastic deformation, the difference between the springback coefficient for both orientations becomes smaller and smaller (Figure 4). The difference in the character of the springback phenomenon of cold rolled sheets is due to the crystallographic structure of the material [7]. The cold rolling of the sheets produces the directional change in the deformation of material microstructure, i.e., the grains are elongated in the direction of the cold rolling process. This results in different material properties of the material rolled, which is explained by planar anisotropy when it gives various flow strengths in various directions in the plane of the sheet metal. Thus, the material is more pronounced to plastic deformation when elongated grains are oriented parallel to the bending plane. Moreover, as shown by Albrut and Barbie [39] in the case of the transverse direction, a significant quantity of deformation energy is used to change the crystallographic orientation of grains.

### 4.2. ANN-Based Springback Prediction

The selection of input variables is a critical element of neural network design. The feature selection analysis is carried out using GA, which has been built in the Statistica program. GAs are global optimisation techniques that work very well with nonlinear problems because of their ability to search a large number of combinations (in this case, sets of input variables) in order to find the best solution. Use of GAs is particularly beneficial in situations where there are correlations between the optimised variables, and the elimination of their interactions must be taken into account when eliminating them. GAs are models for the development of chromosome populations. In the classical version of the algorithm, the chromosome is understood to be a chain of binary values of a certain length. The way the genetic algorithm works when operating consists of the following stages:

1. Adopting a method of coding the real parameters of the adaptation function in the form of a chromosome (interconnected binary numbers representing subsequent parameters which form a chromosome).
2. Determining the form of the adaptation function.
3. Random selection of starting points—the process of searching for the optimal set of parameters begins with the random selection of starting points.
4. Selection of chromosomes for a new population—the selection of individuals for the new population is carried out according to the roulette wheel rule in which each individual corresponds to a segment of a circle of a size proportional to the value of the adaptation function, and then a random point is selected on the wheel and the chromosome to which the randomly drawn part of the circle corresponds is transferred to the new population. This process is repeated until the number of chromosomes in the new population is equal to the number of chromosomes in the old population.
5. Genetic operators are used for individuals from the new population, the most common of which are: crossover and mutation.

Many ANN experiments were carried out with different numbers of neurons in the hidden layer. The evaluation of a set of input variables by the genetic algorithm is made on the basis of the error achieved for the training set. To the network, error is added as the so-called penalty component, which is the product of the defined coefficient (unit penalty) and the number of variables included. Defining the unit penalty expresses the desire to minimise the set of input variables. The analyses were conducted for a unit penalty in the range of δ = 0.0005-0.01 (Table 2). In this analysis “Yes” means that specific parameter carries out the important information which affects the value of springback coefficient Ks. If the specific parameters do not significantly affect the parameter on the output of ANN, then it may be ignored (“Ignore”). The results of future selection carried out in the Statistica program are two-stated, and the specific parameter is important or not important.

**Figure 5:**
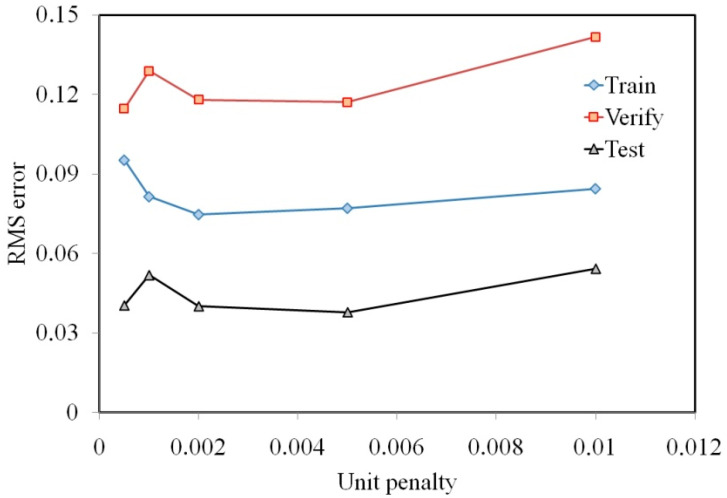
The effect of the unit penalty of the RMS error.

The values of the coefficients of smoothing and of crossover were equal to 0.3. Based on the variables selected by GA, ANN experiments to determine RMS error for the training set were performed. The results of the computations made in Statistica program are shown in Figure 5. The RMS error for the training set, which is represented by the greatest number of training data, reaches a minimum for δ = 0.002. Increasing the unit penalty causes a slight increase in the RMS error for the training set, however the RMS error to verify and test sets increase more significantly. So, further analyses were conducted for MLPs consisting of five neurons in the input layer corresponding to punch bend depth under load f_l_, yield stress σ_y_, strain hardening coefficient K, strain hardening exponent n, and Lankford’s coefficient r.

It was also found that the punch bend depth under load f_l_, strain hardening coefficient K and strain hardening exponent n are not susceptible to the unit penalty. It confirms that these parameters carry important information. The Young’s modulus and ultimate tensile stress are the variables that do not have a significant effect on the coefficient of springback. 

**Figure 6:**
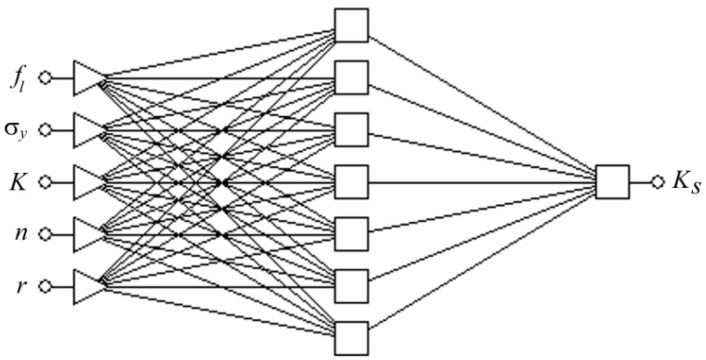
The architecture of the MLP 5:5-7-1:1 network.

Globally, the objective of the analyses of MLPs was to find the network architecture that ensures the smallest value of standard deviation (SD) ratio in connection with a high value of Pearson’s correlation coefficient R [13]. With the help of the Network Designer built in Statistica, a number of network models were established with different numbers of neurons in the hidden layer. The quality of the MLPs was given a preliminary validation by Network Designer and a set of three networks (MLP 5:5-6-1:1, MLP 5:5-7-1:1 and MLP 5:5-9-1:1) with the highest performance were selected for further analysis and the learning process. The highest quality specified in the Statistica program with the performance parameter was characterised by an MLP 5:5-7-1:1 network with five neurons in the input layer and one neuron in the output layer and seven neurons in the hidden layer (Figure 6).

**Figure 7:**
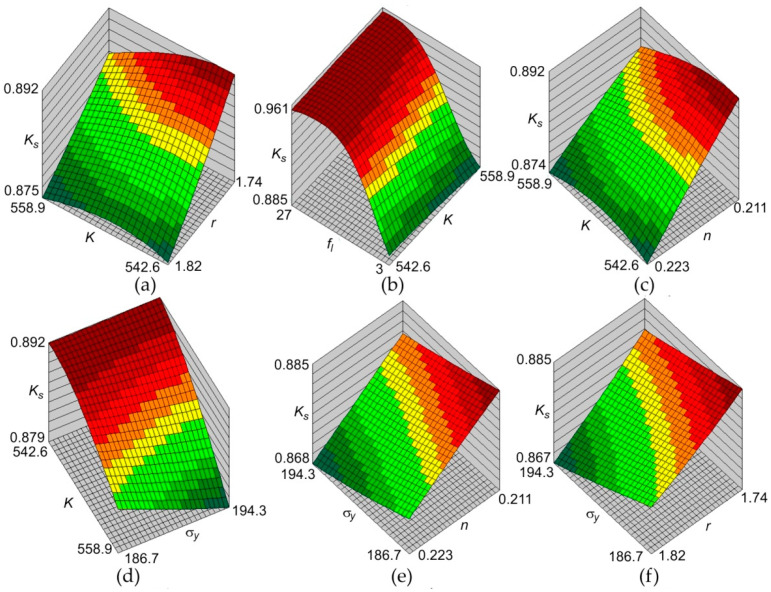

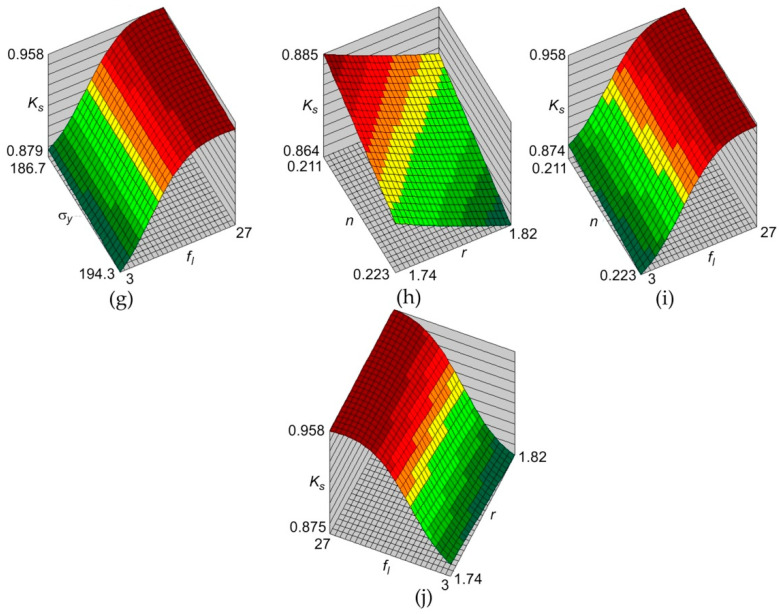
The effect of combinations of (**a**) *K* and *r*, (**b**) *F_l_* and *K*, (**c**) *K* and *n*, (**d**) *K* and *σ_y_*, (**e**) *σ_y_* and *n*, (**f**) *σ_y_* and *r*, (**g**) *σ_y_* and *f_l_*, (**h**) *n* and *r*, (**i**) *n* and *f_l_* and (**j**) *f*_l_ and *r* on the value of springback coefficient *K_s_*.

The surface responses of the MLP 5:5-7-1:1 network are shown in Figure 7. It is clear that increasing punch bend depth under load f_l_ leads to an increase in the predicted springback coefficient K_s_. On the contrary, the predicted value of Ks coefficient decreases with increasing of yield stress σ_y_, strain hardening exponent n, and Lankford’s coefficient r.

The problems relating to the occurrence of local minima as a function of error and the problems associated with the need to make decisions about the size of the network in practice entail the need to carry out a series of experiments with a large number of networks. Each of these must be taught repeatedly in order to avoid learning being falsely stopped by a local minimum. Each of these networks is separately trained and independently assessed in order to select the network that can be considered optimal. The most important information taken into account when assessing the MLPs is the RMS error value for training and validation sets. When assessing the ANN model, special attention should be paid to the SD ratio and the correlation coefficient [38]. Table 3 presents selected statistics of MLPs including the RMS error separately for the training (T) and validation (V) sets. From the point of view of network quality, the smallest RMS error value for a training set is characterised by the network trained with the Quasi-Newton and Lavenberg–Marquardt algorithms. RMS error values for both networks trained by QN and LM algorithms are very similar for all the network architectures tested. The operation of the LM algorithm, combining the gradient descent method and Newton’s algorithm, is based on the assumption that the real function modelled by the ANN that represented the input signal to the output signal is linear. During operation of LM algorithm, it selects a method that gives better results at a given moment by trying hypothetical linear approximation and, if it does not give positive results, it goes back to the gradient descent method. The assumption that the function represented the output signal is linear enables analytical determination of the minimum of error function, but because the real function is not linear, the determined minimum value is not necessarily the actual minimum. The disadvantage of the LM algorithm is finding local minima when the influence of the gradient descent algorithm is becoming more dominated.

The network trained with the BP algorithm (MLP 5:5-7-1:1) has the best statistics, i.e., the highest value of correlation with the lowest value of SD ratio. For each network, the RMS error for the training set was less than the RMS error determined for the validation set. This is due to the different number of learning sets on the basis of which the network gains the ability to generalise data.

Sensitivity analysis allowed the importance of individual input signals in assessing the quality of the network to be determined. MLPs trained by the BP, CG and LM algorithms definitely favour punch bend depth under load f_l_ as the most important variable affecting the springback coefficient K_s_ (Table 4). The second in order of importance is the strain hardening coefficient K, which, on average to the highest degree, was preferred by networks that also learned with the BP, CG and LM algorithms. The anisotropic coefficient r has been recognised by most algorithms as the least important parameter. This does not mean, however, that this parameter does not provide enough information for network input. Probably, this parameter interacts with other input parameters and only then is an information carrier, as demonstrated by the analysis of future selection conducted using GA.

The correlation coefficient of the values of springback coefficient presented at the network output and the values obtained as a result of the training algorithm was between 0.962 and 0.972. At the same time, the SD ratio value was below 0.269. The value of the SD ratio is much smaller than 1, so the MLPs present good performance. Considering that the experimental set consists of only 54 training sets, the quality of the MLP models obtained is acceptable.

**Figure 8:**
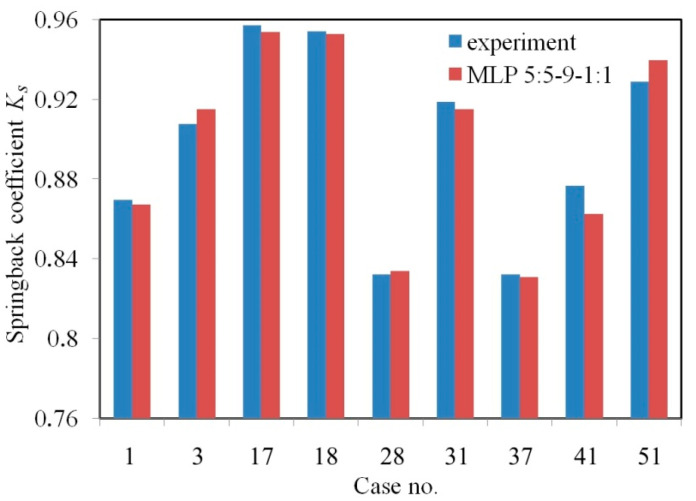
Comparison of the prognostic capacity of the MLP 5:5-9-1:1 trained using the Quasi-Newton (QN) algorithm with the experimental values of springback coefficient *K_s_*.

The forecasting of MLP 5:5-9-1:1 is presented in Figure 8 and Table 5 based on a randomly selected 20% of cases that were assigned for the test set that was not presented during the training process. The high value of the correlation coefficient with the low value of the MSE errors indicates good prediction properties of this neural network. This is reflected in the good matching of the values of springback coefficient determined by the ANN with the experimental data represented by the test set.

## 5. Conclusions

This paper presents the results of the prediction of springback in cold-rolled anisotropic steel sheets by using a coupled ANN and GA approach. The following conclusions are drawn from the research:

1. The specimens cut transverse to the rolling direction exhibit lower values of springback coefficient *K_s_* compared to the specimens cut along the rolling direction. The difference between the springback coefficients measured for both directions does not exceed 2.3%.
2. An increase in the bend angle *γ_l_* leads to a nonlinear increase in the springback coefficient *K_s_*. The higher the bend angle *γ_l_*, the smaller the difference between the springback coefficients *K_s_* measured for both sample orientations.
3. The Young’s modulus *E* and ultimate tensile stress *σ_m_* are variables which have no significant effect on the coefficient of springback.
4. The smallest RMS error value for a training set is seen in the network trained with the QN and LM algorithms. RMS error values for both networks trained by these algorithms are very similar for all the network architectures tested.
5. The network trained with the BP algorithm (MLP 5:5-7-1:1) had the highest value of correlation coefficient in connection with the lowest value of SD ratio.
6. For each network, the RMS error for the training set was less than the RMS error determined for the validation set. This is due to the different number of learning sets on the basis of which the network gains the ability to generalise data.
7. The MLP trained by the BP, CG and LM algorithms favoured the punch bend depth under load *f_l_* as the most important variable affecting the springback coefficient *K_s_*.
8. It was found that the anisotropic coefficient *r* was the least important parameter.

## Figures and Tables

**Table 1:** Mechanical properties of the DC04 steel sheets ^1^.

Orientation of Specimen	*E*, MPa	*σ_y_*, MPa	*σ_m_*, MPa	*K*, MPa	*n*	*r*
0°	1.98·10^5^	186.7	324.1	558.9	0.211	1.74
90°	1.92·10^5^	194.3	318.6	542.6	0.223	1.82

^1^*E*—Young’s modulus, *σ_y_*—yield stress, *σ_m_*—ultimate tensile stress, *K*—strain hardening coefficient, *n*—strain hardening exponent, *r*—Lankford’s coefficient.

**Table 2:** The results of future selection analysis at δ = 0.0005 to 0.01.

Parameter	*f_l_*	*E*	*σ_y_*	*σ_m_*	*K*	*n*	r
Useful at δ = 0.0005	Yes	Yes	Ignore	Ignore	Yes	Yes	Ignore
Useful at δ = 0.001	Yes	Ignore	Yes	Ignore	Yes	Yes	Yes
Useful at δ = 0.002	Yes	Ignore	Yes	Ignore	Yes	Yes	Yes
Useful at δ = 0.005	Yes	Yes	Ignore	Ignore	Yes	Yes	Yes
Useful at δ = 0.01	Yes	Ignore	Yes	Ignore	Yes	Yes	Ignore

**Table 3:** Effect of the learning algorithm on the statistics of Multilayer Perceptrons (MLPs).

	BP				CG				QN				LM			
MLPs	RMS(T)	RMS(V)	SD Ratio	Correlation	RMS(T)	RMS(V)	SD Ratio	Correlation	RMS(T)	RMS(V)	SD Ratio	Correlation	RMS(T)	RMS(V)	SD Ration	Correlation
MLP 5:5-6-1:1	0.085	0.129	0.270	0.962	0.769	0.113	0.269	0.963	0.068	0.128	0.240	0.970	0.068	0.126	0.239	0.971
MLP 5:5-7-1:1	0.084	0.107	0.254	0.967	0.077	0.118	0.265	0.964	0.068	0.129	0.237	0.971	0.067	0.129	0.235	0.972
MLP 5:5-9-1:1	0.112	0.165	0.262	0.965	0.073	0.124	0.257	0.967	0.067	0.145	0.235	0.972	0.068	0.129	0.239	0.971

**Table 4:** Sensitivity analysis of the test MLPs.

	BP					CG					QN					LM				
Test MLPs	*f_l_*	*σ_y_*	*K*	*n*	*r*	*f_l_*	*σ_y_*	*K*	*n*	*r*	*f_l_*	*σ_y_*	*K*	*n*	*r*	*f_l_*	*σ_y_*	*K*	*n*	*r*
MLP 5:5-6-1:1	•••••	••	•••	••••	•	•••••	•	•••	••••	••	••••	•••	•••••	••	•	•••••	•••	••••	•	••
MLP 5:5-7-1:1	•••••	•••	••••	••	•	••••	•••••	•••	••	•	•	•••••	••	•••	••••	••••	•	•••	•••••	••
MLP 5:5-9-1:1	••••	•	•••••	••	•••	•••••	••	••••	•••	•	•••	••••	•	•••••	••	•••••	•	••••	•••	••

(•••••—signal the most important, •—signal the least important).

**Table 5:** Quantitative comparison of the springback coefficients Ks determined experimentally and by MLP 5:5-9-1:1.

Case	Input Parameter					Springback Coefficient *K_s_*	
	*f_l_*, mm	*σ_y_*, MPa	*K*	*n*	*r*	ANN	Experiment
1	3	186.7	558.9	0.211	1.74	0.867	0.869
13	12	186.7	558.9	0.211	1.74	0.914	0.907
17	24	186.7	558.9	0.211	1.74	0.953	0.956
18	27	186.7	558.9	0.211	1.74	0.952	0.954
28	3	194.3	542.6	0.223	1.82	0.834	0.832
31	12	194.3	542.6	0.223	1.82	0.914	0.918
37	3	194.3	542.6	0.223	1.82	0.831	0.832
41	15	194.3	542.6	0.223	1.82	0.862	0.876
51	18	194.3	542.6	0.223	1.82	0.939	0.928
